# Conservation Study of Imprinted Genes in Maize Triparental Heterozygotic Kernels

**DOI:** 10.3390/ijms232315424

**Published:** 2022-12-06

**Authors:** Xiaomei Dong, Haishan Luo, Jiabin Yao, Qingfeng Guo, Shuai Yu, Xiaoyu Zhang, Fenghai Li, Yanye Ruan, Weiwei Jin, Dexuan Meng

**Affiliations:** 1 College of Bioscience and Biotechnology, Shenyang Agricultural University, Shenyang 110866, China; 2 Shenyang City Key Laboratory of Maize Genomic Selection Breeding, Shenyang 110866, China; 3 College of Agronomy, Shenyang Agricultural University, Shenyang 110866, China; 4 State Key Laboratory of Plant Physiology and Biochemistry, National Maize Improvement Center, Key Laboratory of Crop Heterosis and Utilization, the Ministry of Education, College of Agronomy and Biotechnology, China Agricultural University, Beijing 100193, China; 5 Department of Agronomy, College of Agriculture & Resources and Environmental Sciences, Tianjin Agricultural University, Tianjin 300392, China

**Keywords:** maize, allelic expression, imprinted genes, conservation, kernel development

## Abstract

Genomic imprinting is a classic epigenetic phenomenon related to the uniparental expression of genes. Imprinting variability exists in seeds and can contribute to observed parent-of-origin effects on seed development. Here, we conducted allelic expression of the embryo and endosperm from four crosses at 11 days after pollination (DAP). First, the F1 progeny of B73(♀) × Mo17(♂) and the inducer line CAU5 were used as parents to obtain reciprocal crosses of BM-C/C-BM. Additionally, the F1 progeny of Mo17(♀) × B73(♂) and CAU5 were used as parents to obtain reciprocal crosses of MB-C/C-MB. In total, 192 and 181 imprinted genes were identified in the BM-C/C-BM and MB-C/C-MB crosses, respectively. Then, by comparing the allelic expression of these imprinted genes in the reciprocal crosses of B73 and CAU5 (BC/CB), fifty-one Mo17-added non-conserved genes were identified as exhibiting imprinting variability. Fifty-one B73-added non-conserved genes were also identified by comparing the allelic expression of imprinted genes identified in BM-C/C-BM, MB-C/C-MB and MC/CM crosses. Specific Gene Ontology (GO) terms were not enriched in B73-added/Mo17-added non-conserved genes. Interestingly, the imprinting status of these genes was less conserved across other species. The cis-element distribution, tissue expression and subcellular location were similar between the B73-added/Mo17-added conserved and B73-added/Mo17-added non-conserved imprinted genes. Finally, genotypic and phenotypic analysis of one non-conserved gene showed that the mutation and overexpression of this gene may affect embryo and kernel size, which indicates that these non-conserved genes may also play an important role in kernel development. The findings of this study will be helpful for elucidating the imprinting mechanism of genes involved in maize kernel development.

## 1. Introduction

Imprinting is an epigenetic phenomenon in which the expression of a subset of genes is dependent on the parent of origin [1]. In 1970, *R1* was discovered in maize as a maternally expressed gene (MEG) based on the observation that fully colored kernels were produced when both the *R* and *B* alleles were inherited from the maternal parent but not from the paternal parent [2,3,4]. With the wide application of high-throughput sequencing technology, the efficiency of identifying imprinted genes has been greatly improved. Transcriptomic analysis during plant development in hybrids is an effective approach to detecting imprinted genes in flowering plants [5]. Hundreds of imprinted genes have been reported in Arabidopsis, maize, rice and sorghum through this method [6,7,8,9,10,11,12,13,14,15,16,17,18]. Imprinted genes play important roles in plant development, especially in seed development. For example, *defective 18(de18),* a paternally expressed gene (PEG), was recently reported to play a significant role in endosperm development [19]. In Arabidopsis, an MEG, *FORMIN HOMOLOGY5(FH5)*, was reported to be responsible for endosperm cellularization [20].

There are three hypotheses used to explain the evolution of imprinted genes [21]: kinship theory [22,23,24], sexual antagonism theory [25] and maternal–offspring coadaptation theory [26,27,28]. Among these, the theory of parent–offspring conflict, also known as kinship theory, suggests that genomic imprinting is driven by competition in the endosperm between paternal and maternal alleles for the allocation of resources from mother to offspring. This theory has been supported by much evidence [16,24,29,30]. It is inferred that the same locus would be selected between two closely related species because of the shared conflict between parents. Between plant species, the conservation of imprinting was shown to be lower than that within species. In rice, 168 imprinted genes were discovered using a reciprocal cross between the 9311 (Indica) and the Nipponbare (Japonica) cultivars [31], and 257 imprinted genes were found using reciprocal crosses between the Longtefu (Indica) and the 02428 (Japonica) cultivars. In rice, 13 out of 14 imprinted genes selected from cultivated plants were also imprinted in reciprocal crosses between wild rice and cultivated rice [32]. Moreover, 46% of these genes were commonly detected in at least two sets of four crosses. In Arabidopsis, 208 and 123 imprinted genes were detected from the same reciprocal crosses between the Columbia (Col) and Landsberg erecta (Ler) ecotypes by two research groups [22,33], and only 20 were common across studies. Additionally, in maize, 179 and 100 imprinted genes were detected from the same cross between Mo17 and B73 by two research groups [23,34]. Among these genes, only 56 were the same, and the researchers inferred that the regulatory mechanism of imprinting was conserved between closely related species. Within plant species, most imprinted genes exhibit conserved imprinting [13,35,36,37]. However, only some imprinted genes exhibit interspecies imprinting variability. Interestingly, the epiallelic variation in *HOMEDOMAIN GLABROUS3* (*HDG3*) can lead to variation in seed development phenotypes [13].

Recent studies have indicated that, except for genetic variations, epigenetic variations potentially occur in differential gene expression and growth vigor in plant hybrids. In maize roots and shoots, the global distribution patterns of epigenetic components between parents and hybrid offspring have been analyzed [38,39]. Extensive variations in DNA methylation, histone modifications and siRNA transcriptional levels were observed in parents and hybrids from shoots and roots [40]. These diverse epigenetic variations potentially play important roles in altering genome activity and gene expression in different organs of hybrid offspring compared with their parents by modulating chromatin states. DNA methylation, histone modifications and siRNA transcriptional levels make an important contribution to the epigenetic regulation of genomic imprinting. Hence, it is not clear whether some genes changed their imprinting expression when introducing a hybridization process from outside the original background. In this study, we compared the allelic expression of imprinted genes identified in BM-C/C-BM and MB-C/C-MB crosses with the imprinted genes detected in BC/CB and MC/CM crosses. We discovered 11 high-confidence examples of allele-specific imprinting. Finally, the gene phenotypic analysis of one non-conserved PEG verified that the mutation of this gene made the embryo and kernel smaller than those of the wild type, and the overexpression of this gene enlarged the embryo and kernel area. Our results contribute to understanding the allelic variation in imprinted genes and the function of imprinted genes in maize kernel development.

## 2. Results

### 2.1. Analysis of Gene Expression and Identification of Imprinted Genes in Reciprocal Crosses

To understand the differences in gene expression in the early embryo and endosperm, we performed RNA sequencing (RNA-seq) analysis on the immature embryo and endosperm from reciprocal crosses between the inducer line CAU5 and hybrid lines (BM or MB) at 11 DAP. Two reciprocal crosses were used and were designated as BM-C/C-BM, representing (B73 × Mo17) × CAU5/CAU5 × (B73 × Mo17), and MB-C/C-MB, representing (Mo17 × B73) × CAU5/CAU5 × (Mo17 × B73) (Figure S1A). Considering that haploids may have been produced in the hybrid crosses (BM-C and MB-C) when the CAU5 inducer line was used as the male parent, we not only selected the diploid embryo of each cross for further analysis but also extracted three replicates from multiple samples each for the diploid embryo and triploid endosperm. By combining correlation analysis data between these samples, we found that the correlation of the three biological replicate samples in each combination of each tissue was greater than 0.97, which indicated that the gene expression data were highly reproducible (Figure S1B). Illumina sequencing generated an average of 13.84 M paired-end clean reads from each biological replicate (Table S1), which were aligned to the reference genome (see Section 4).

**Figure 1:**
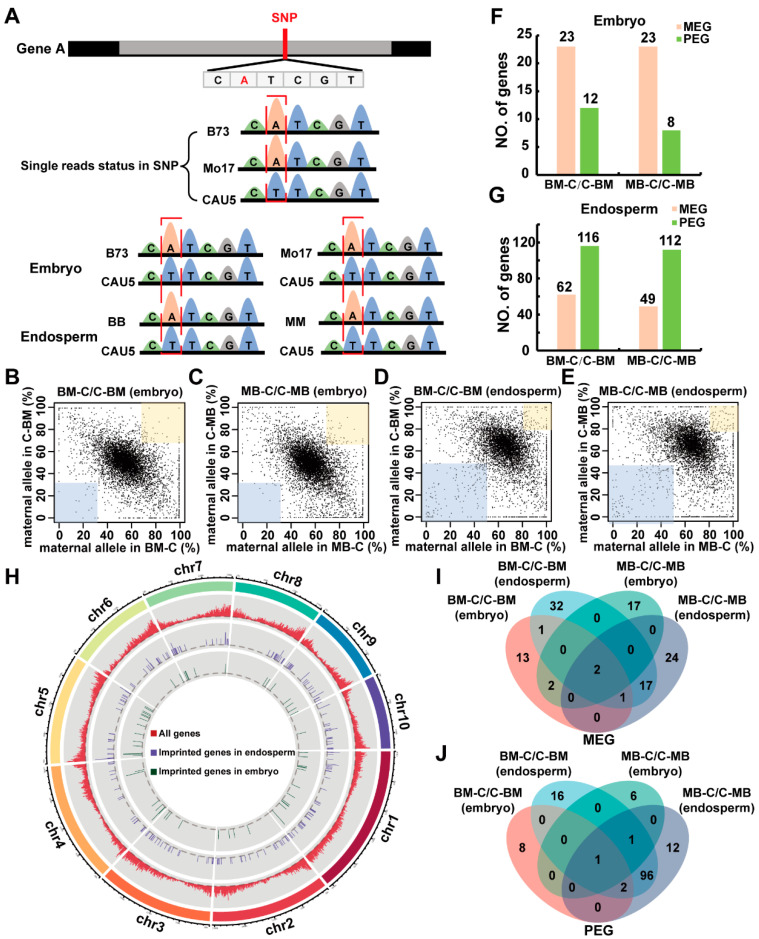
Discovery of imprinted genes detected in the embryo and endosperm of two reciprocal hybrids (BM-C/C-BM and MB-C/C-MB). (**A**) The SNP locus used for distinguishing the parental reads origin. (**B**–**E**) The proportion of parental transcripts in two reciprocal hybrids (BM-C/C-BM, MB-C/C-MB). (**F**,**G**) The number of imprinted genes identified in the embryo and endosperm. Pink and green rectangles represent the number of MEG and PEG in the embryo (**F**) and endosperm (**G**). (**H**) Chromosomal distribution of imprinted genes identified in the embryo and endosperm from BM-C/C-BM and MB-C/C-MB crosses. The red, purple and dark-green lines represent, respectively, all genes, imprinted genes identified in the endosperm and imprinted genes identified in the embryo. (**I**,**J**) Comparison of MEG and PEG numbers identified in the embryo and endosperm from BM-C/C-BM and MB-C/C-MB crosses.

Since three inbred lines were used in the reciprocal cross tests, an SNP locus was selected in our plant materials to distinguish the parental read origin. This locus was not only different between B73 and CAU5 but was also the same in B73 and Mo17 (Figure 1A). To identify genes that showed significant bias toward the maternal or paternal origin of transcription, we generated a scatter plot to show the relative transcription output from the maternal and paternal alleles of each gene, with more than 20 allelic reads in the embryo and endosperm transcriptome data (Figure 1B–E). As shown in Figure 1F,G, using the threshold of 2:1 in the embryo and endosperm (see methods), we found 35 (23 MEGs and 12 PEGs) and 178 (62 MEGs and 116 PEGs) imprinted genes in the embryo and endosperm from the BM-C/C-BM hybrid combination, respectively, and 31 (23 MEGs and 8 PEGs) and 161 (49 MEGs and 112 PEGs) imprinted genes in the embryo and endosperm from the MB-C/C-MB hybrid combination, respectively (Table S2).

We next examined the chromosomal location of the imprinted genes detected in all the pairs of reciprocal crosses (Figure 1H) and found that chromosome 1 had the greatest number of imprinted genes (51 genes), while both chromosomes 6 and 8 had the lowest number (17 genes). We also scanned the genome for candidate clusters containing at least two adjacent imprinted transcripts within a region of 1 Mb; two imprinted genes fell into one cluster in the embryo, and two imprinted genes fell into one cluster in the endosperm (Table S3). To further analyze the tissue-specific genes between the embryo and endosperm in the three crosses, we counted the number of imprinted genes detected in each cross. We found that 192 and 181 imprinted genes were detected in BM-C/C-BM and MB-C/C-MB, respectively, and the majority were specifically expressed in these tissues (Figure 1I,J).

### 2.2. Conservation and GO Analysis of the Non-Conserved Genes

To study whether the imprinting status of some genes changed when another parent was added to the hybridization crosses, we compared the difference in imprinting status among the BC and CB crosses (designations representing B73 × CAU5 and CAU5 × B73), the MC and CM crosses (representing Mo17 × CAU5 and CAU5 × Mo17), the BM-C/C-BM crosses and the MB-C/C-MB crosses, and we denoted them as non-conserved genes.

**Figure 2:**
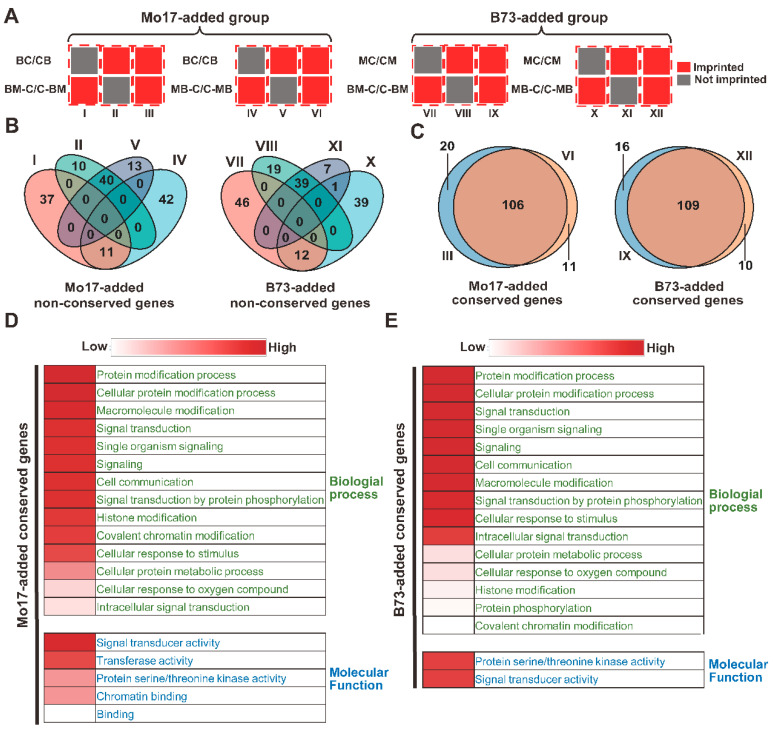
Conservation and GO analysis of the Mo17-added and B73-added non-conserved genes. (**A**) Classification of the genes belonging to the B73-added group and Mo17-added group. The Mo17-added group was divided into six sets: I, II, III, IV, V and VI. The B73-added group was divided into six sets: VII, VIII, IX, X, XI and XII. (**B**) Overlap analysis of the Mo17-added non-conserved genes and B73-added non-conserved genes. I, II, IV and V refer to four sets of Mo17-added groups in Figure 2A. VII, VIII, XI and X refer to the four sets of B73-added groups in Figure 2A. (**C**) Overlap analysis of the Mo17-added conserved genes and B73-added conserved genes. III and VI refer to the two sets of Mo17-added groups in Figure 2A. IX and XII refer to the two sets of B73-added groups in Figure 2A. (**D**) GO analysis of the Mo17-added conserved genes. (**E**) GO analysis of the B73-added conserved genes.

In this comparison, two groups of genes were used to analyze whether the imprinting status difference changed. The first was the “Mo17-added” group, which represented six sets of genes (Figure 2A): I: imprinted in BM-C/C-BM crosses but not imprinted in BC/CB crosses; II: imprinted in BC/CB crosses but not imprinted in BM-C/C-BM crosses; III: imprinted in both BM-C/C-BM crosses and BC/CB crosses; IV: imprinted in MB-C/C-MB crosses but not imprinted in BC/CB crosses; V: imprinted in BC/CB crosses but not imprinted in MB-C/C-MB crosses; and VI: imprinted in both MB-C/C-MB crosses and BC/CB crosses. Among the 153 genes in sets I, II, IV and V, 51 genes overlapped between these four sets, and we identified the 51 genes as “Mo17-added non-conserved genes” (Figure 2B). Then, in the “B73-added” group, genes were also divided into six sets, and 51 of the 163 genes overlapped between sets VII, VIII, X and XI. Similarly, we identified these genes as “B73-added non-conserved genes” (Figure 2B). We further analyzed the interspecific conservation of Mo17-added non-conserved genes and B73-added non-conserved genes. Only 12% and 10% of the Mo17-added and B73-added non-conserved genes were conserved imprinting in intraspecies, respectively. For Mo17-added and B73-added conserved genes, 36% were conserved imprinting in rice and Arabidopsis (Table S4).

In contrast, we also compared the genes whose imprinting status did not change when another parent was added to the hybridization crosses and denoted them as conserved genes. As a result, 106 Mo17-added conserved genes (genes overlapped between sets III and VI) and 109 B73-added conserved genes (genes overlapped between sets IX and XII) were identified. Surprisingly, nearly 90% of the Mo17-added conserved genes were overlapped with B73-added conserved genes (Figure 2C). Furthermore, these genes were also more conserved within species. For Mo17-added conserved genes, 49% of the genes were conserved within sorghum (28%) and rice (25%). Similarly, for B73-added conserved genes, 45% of the genes were conserved in sorghum and rice, and more genes showed conserved imprinting in sorghum (27%) and rice (19%) (Table S4).

To study the potential function of the above-conserved and non-conserved imprinted genes in maize development, especially in kernel development, GO analysis was performed (Figure 2D,E). First, we focused on the pathways enriched in the B73-added conserved group and Mo17-added conserved group, and nearly 90% of the enriched pathways in the two groups were identical. Signal transduction and protein modification processes, especially histone modification in the biological process category, were both highly enriched in the two groups. In the molecular function category, kinase activity and signal transducer activity were highly enriched. However, neither category showed significant enrichment in the genes of B73-added or Mo17-added non-conserved groups.

### 2.3. Cis-Element Distribution, Expression Pattern and Subcellular Location of Proteins from the Conserved and Non-Conserved Imprinted Genes

**Figure 3:**
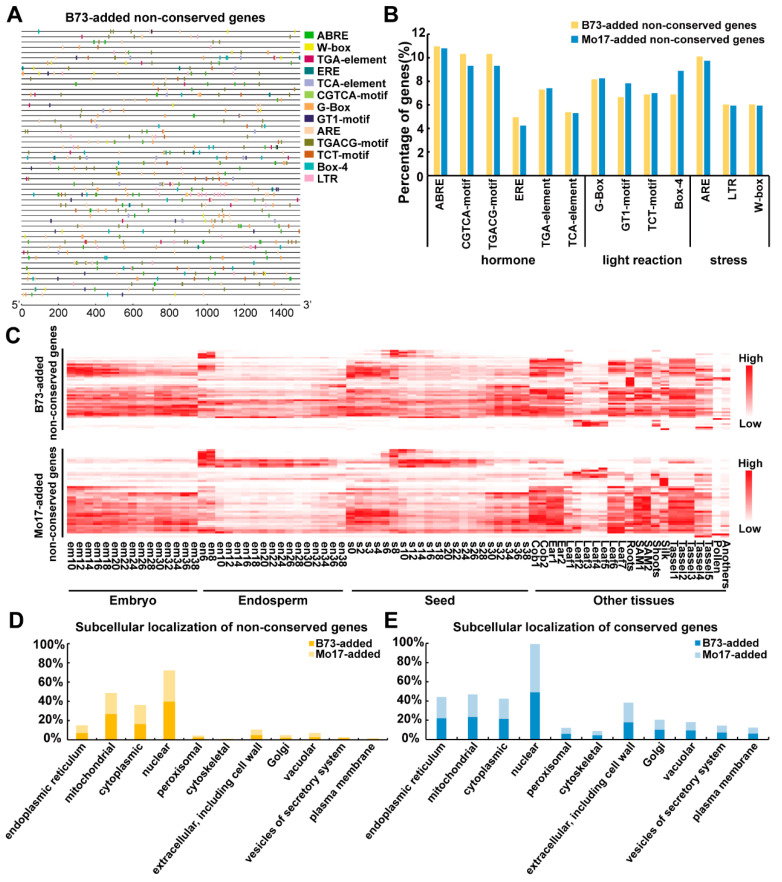
The cis-element distribution, gene expression level and subcellular localization of proteins encoded by conserved and non-conserved imprinted genes of the B73-added group and the Mo17-added group. (**A**) The cis-element distribution in the promoter sequence of the 51 non-conserved imprinted genes detected in the B73-added group; distribution of cis-acting elements related to hormones, light and stress in the promoter sequences of the 51 genes. Putative ABRE, W-box, TGA-element, ERE, TCA-element, CGTCA-motif, G-box, GT1-motif, ARE, TGACG-motif, TCT-motif, Box 4 and LTR core sequences are represented by different symbols, as indicated in the legend on the right. The 1.5 kb sequences upstream of the initiation codon (ATG) of the imprinted genes were estimated using the scale per 300 bp above. (**B**) The ratio of each cis-element in the two groups of non-conserved genes. Yellow and blue rectangles represent the B73-added and Mo17-added non-conserved groups. (**C**) The expression of the imprinted genes detected in the B73-added and Mo17-added non-conserved groups. (**D**,**E**) The subcellular localization of proteins encoded by non-conserved (**D**) and conserved (**E**) imprinted genes in the B73-added and Mo17-added non-conserved groups. The *Y*-axis represents the percentage of proteins located in each organelle. Yellow indicates proteins encoded by genes in the Mo17-added non-conserved group; dark-yellow indicates proteins encoded by genes in the B73-added non-conserved group; blue indicates proteins encoded by genes in the Mo17-added conserved group; and dark blue indicates proteins encoded by genes in the B73-added conserved group.

To elucidate the conserved and non-conserved imprinted gene regulatory mechanism in response to abiotic or biotic stress in maize, the genomic sequence of the 1.5 kb upstream promoters of the non-conserved and conserved imprinted genes was used to query the Plant Care database to search for cis-regulatory elements (CREs) (Figure 3A and Figure S2, Figure S3 and Figure S4). Thirteen classes of CREs related to hormones (ABRE, CGTCA-motif, TGACG-motif, ERE, TGA-element and TCA-element), light reactions (G-box, GT1-motif, TCT-motif and Box-4) and stress (ARE, W-box and LTR) were detected. The six hormone-related CREs had the largest proportion and were distributed in 51 (51), 48 (44) and 48 (44) non-conserved imprinted genes in the B73-added (Mo17-added) groups (Figure S5). Each predicted CRE existed in at least 20 non-conserved genes (Figure S5), and each gene has at least six CRE distributions (Figure S6, Table S5). We also compared the difference in the cis-element ratios of the non-conserved and conserved imprinted genes in the B73-added group and the Mo17-added group (Figure 3B and Figure S7), and we found that they had nearly the same ratio, which may indicate that these genes share a similar regulatory mechanism. Furthermore, we also compared the difference between the non-conserved and conserved imprinted genes in the B73-added group and the Mo17-added group, and both groups showed differences in G-box elements (Figure S8).

Then, we compared the gene expression levels of the B73-added and Mo17-added conserved and non-conserved genes. More constitutively expressed genes were detected in the conserved group (Figure 3C and Figure S9). The subcellular locations of proteins encoded by non-conserved and conserved imprinted genes in the B73-added and Mo17-added groups were analyzed on the website for GenScript-PSORT II (https://www.genscript.com/psort.html?src=leftbar, accessed on 20 August 2022). The 249 B73-added conserved imprinted genes, 251 Mo17-added conserved imprinted genes, 51 B73-added non-conserved imprinted genes and 51 Mo17-added non-conserved imprinted genes were separated into various subcellular locations. In these four types of genes, more than 40% of the genes were located in the nucleus (Figure 3D,E), which is consistent with the results of previous studies that showed that most imprinted genes were located in the nucleus; moreover, some of them were transcription factors that may play an important role in the kernel development process [41]. In addition, in the B73-added non-conserved genes and Mo17-added non-conserved genes, 20% and 17% of the genes were located in the mitochondria and cytoplasm, respectively. Unlike the above-mentioned non-conserved group of genes, the B73-added and Mo17-added conserved genes were present in all organelles (Figure 3E).

### 2.4. Extremely Non-Conserved Imprinted Genes Detected in Different Species

**Figure 4:**
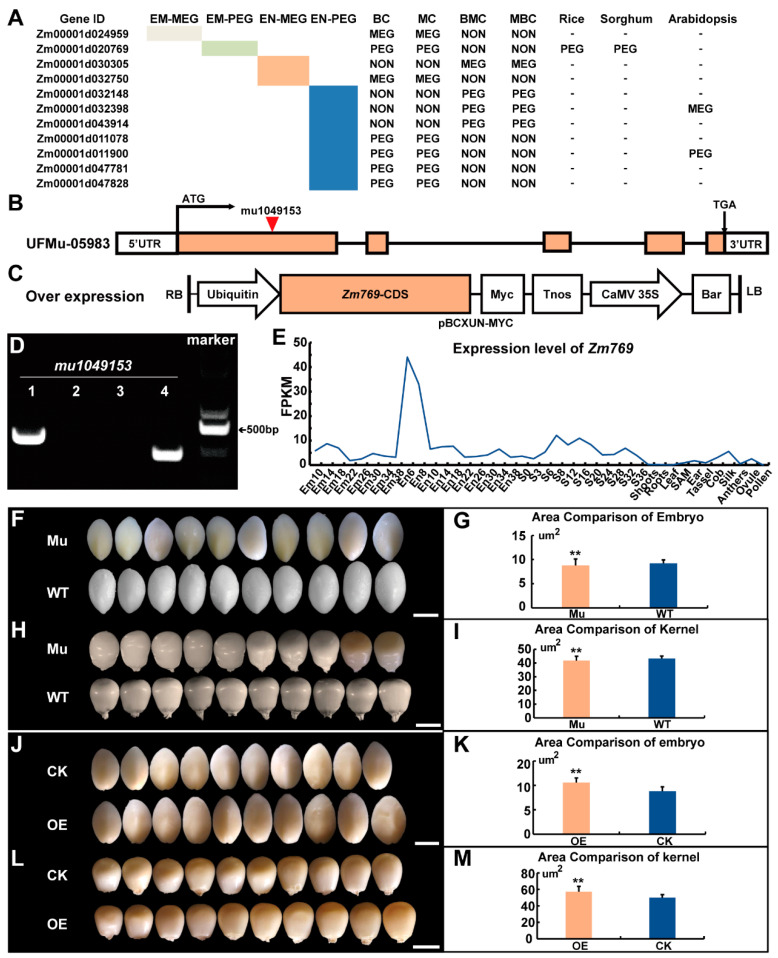
Embryo and kernel phenotypes of the *Zm769* mutant and overexpression lines. (**A**) Conservation of non-conserved imprinted genes across species. (**B**) Locus of mu1049153 insertion in UFMu-05983. (**C**) Carrier structure of overexpression lines of *Zm769*. (**D**) Verification of Mu insertion of the *Zm769* gene: mu1049153. Lanes 1 and 3 represent the wild-type (W22) DNA; lanes 2 and 4 represent the mutant DNA. Lanes 1 and 2 represent samples using the forward and reverse primers from the corresponding gene sequence (Supplemental Table S6); Lanes 3 and 4 represent samples using TIR6 as the forward primer and the same reverse primers as Lanes 1 and 2 (Supplemental Table S6). (**E**) Expression level of *Zm769*. (**F**,**G**) Embryo phenotypes of the mutant line and background line. (**H**,**I**) Kernel phenotypes of the mutant line and background line. (**J**,**K**) Embryo phenotypes of the overexpression line and transgene receptor line. (**L**,**M**) Kernel phenotypes of the overexpression line and transgene receptor line. Significant differences were analyzed by two-tailed Student’s t tests (** *p* < 0.01).

To study whether the imprinted status of some non-conserved genes was stable in both the B73-added and Mo17-added groups, we performed an overlap analysis of 102 non-conserved genes in the two groups (51 genes in the B73-added non-conserved group and 51 genes in the Mo17-added non-conserved group). Eleven genes were analyzed further. We first detected the imprinted status of these 11 genes in our results and found that 1 MEG and 1 PEG were imprinted in the embryo, and that 2 MEGs and 7 PEGs were imprinted in the endosperm. Then, we analyzed the interspecific conservation of these 11 genes in sorghum, rice and Arabidopsis (Figure 4A). Two MEGs and two PEGs were also imprinted in rice (one MEG and two PEG), sorghum (one PEG) and Arabidopsis (one MEG and one PEG).

### 2.5. Phenotype Analysis of the Non-Conserved PEG Zm00001d020769 Transgenic Line

To explore the function of these 11 non-conserved imprinted genes during embryo development in maize, we used gene mutation and overexpression lines for further genotype analysis (Figure 4B,C). First, we searched for mutants of 11 genes in the Maize Genetics COOP Stock Center, and one of them, *Zm00001d020769* (*Zm769*), had a Mu insertion, UFMu-05983, with the mu1049153 insertion in the first exon (Figure 4B). The Mu insertion sequences were verified with two sets of PCRs with gene-specific primers and a Mu-specific primer (Figure 4D, Table S6). *Zm769* was a PEG detected in the embryo and highly expressed in the kernel, which encoded a ubiquitin-specific protease (Figure 4E). Therefore, we focused on comparing the embryo and kernel phenotypes of the mutant lines with the background line to determine whether any abnormal kernel phenotypes occurred during development. The embryos and kernels in mutant lines both showed significant differences compared to those in the wild W22 inbred line (*p* value < 0.01, Student’s test), suggesting an important role for this gene in maize embryo development (Figure 4F–I). Then, we used transgene technology to create an overexpression line (*Zm769*-OE) to further analyze the function of *Zm769*. As shown in Figure 4J–M, at 15 days after self-pollination, the embryo and kernel areas of the *Zm769* overexpression lines were significantly larger than those of the transgenic receptor, which indicated that *Zm769* may participate in the kernel development process.

## 3. Discussion

Cross direction plays an important role in heterotic and kernel development in maize by affecting gene expression in hybrid offspring [11,12,34,40,42,43]. For example, the size of the 301 × 005 embryo was significantly larger than that of the 005 × 301 embryo at 6 DAP [44]. Genomic imprinting refers to the parental-specific expression of a number of genes after fertilization that occurs in the embryo and endosperm. However, the effects of hybridization on genomic imprinting and its influence on kernel development remain elusive. In our results, the imprinted status of 51 genes was changed when introducing a new parent in both the B73-added group and the Mo17-added group. In view of the factors besides environmental impact that affect the imprinting state of genes, different parental backgrounds may be the key factor of the imprinted status difference. As in the crosses BM-C/C-BM and MB-C/C-MB, the B73, Mo17 and CAU5 parents used in the two reciprocal crosses were the same, so the different imprinted statuses of these non-conserved genes may be substantially affected by the hybridization direction of the two parents (BM and MB). Interestingly, the overlap ratio of the imprinted genes detected in BM-C/C-BM and MB-C/C-MB was more than 80%, which not only confirmed the accuracy of our results but also verified the stability of the conserved imprinted genes. Of course, the cross direction seems to have an effect on the allelic expression of imprinted genes. When BM or MB was used as one parent to cross with CAU5, approximately 25% of imprinted genes exhibited differential allelic expression (Figure 1I-J). Hence, by systematically investigating imprinted genes in four crosses, BC/CB, MC/CM, BM-C/C-BM and MB-C/C-MB, we found that the process of hybridization and cross direction have effects on genomic imprinting. Recently, global epigenetic variations and their potential association with altered gene expression in hybrids have been extensively discussed [45,46,47]. Therefore, in future work, there will be a need to comprehensively determine epigenetic variations during hybridization and their association with imprinting variations.

In addition to the conserved imprinted genes identified previously in plants [8,9,12,34,36], we identified 137 and 135 conserved imprinted genes in our results. GO term analysis revealed that signal transduction, protein modification processes and, especially, histone modification in the biological process category were highly enriched in the identified genes. Additionally, the molecular function category was highly enriched in genes related to kinase activity and signal transducer activity (Figure 2). Unfortunately, none of the categories was significantly enriched in either the 51 B73-added non-conserved group of genes nor the 51 Mo17-added non-conserved group of genes. To explore whether non-conserved imprinted genes are a byproduct of gametogenesis epigenetic reprogramming, genotypic and phenotypic analyses of one non-conserved PEG were performed. The mutation of this gene affects embryo and kernel size, which indicates that these non-conserved genes may also play an important role in kernel development. Therefore, further investigations of the extent of parental bias related to the function of B73-added/Mo17-added non-conserved imprinted genes should be conducted in the future.

## 4. Materials and Methods

### 4.1. Plant Materials

The hybrid lines B73(♀) × Mo17(♂) and Mo17(♀) × B73(♂) were obtained from the inbred lines B73 and Mo17 in the summer of 2020 at the experimental station of Shenyang Agriculture University in Shenyang, Liaoning. Then, in the summer of 2021, the inducer line CAU5 and these two hybrid lines were used as the parental lines for reciprocal crosses. The ears and tassels of the three lines were bagged with kraft paper one day prior to pollination. The next day, each paper bag was patted to collect pollen from one parent, which was used to pollinate the ear of the other parent. After 11 days, the ears of four reciprocal crosses (BM-C/C-BM, MB-C/C-MB) were collected. In this study, BM-C/C-BM represents the crosses between (B73 × Mo17) × CAU5 and CAU5 × (B73 × Mo17), and MB-C/C-MB represents the reciprocal crosses between (Mo17 × B73) × CAU5 and CAU5 × (Mo17 × B73).

### 4.2. Library Construction for RNA-Seq

The embryo and endosperm samples were isolated using a Quick RNA Isolation Kit (Huayueyang Biotechnology Company, Beijing, China). mRNA library construction and sequencing were conducted following the Illumina manufacturer instructions. Total RNA was extracted as input material for the RNA sample preparations. The NEB Next^®^ Ultra TM RNA Library Prep Kit from Illumina^®^ (New England Biolabs (NEB), Ipswich, MA, USA) was used to generate mRNA libraries. High-throughput mRNA sequencing was performed using the Illumina NovaSeq 6000 platform, and 150 bp paired-end reads were generated for each library. An average of 4.2 Gb of data for each replicate was obtained and used for the following analyses, providing sufficient sequencing depth for the imprinting analysis.

### 4.3. Read Mapping, Gene Expression Analysis and SNP Calling

First, clean reads were mapped to the B73 reference genome (Version 4) using HISAT2 software with default parameters [48]. Cufflinks software (V2.2.1) was used to estimate the normalized gene expression values (FPKM) [49]. The calculated log_2_ (FPKM + 1) values were used to analyze the correlation coefficient between replicates. Hierarchical clustering analysis was performed on the relative expression value by setting the parameters average linkage and Euclidean distance using MeV (http://www.tm4.org/mev.html, accessed on 20 July 2021). For a gene in a special sample, the relative expression value was the FPKM normalized by the maximum FPKM value of the gene over all samples. Based on the clustering results, MEGs (or PEGs) primarily expressed in endosperm as a subgroup were obtained, and the rest of the MEGs (or PEGs) were assigned to the constitutively expressed subgroup.

Resequencing reads of B73, Mo17 and CAU5 inbred lines were downloaded from NCBI (SRR12415217, SRR12415218 and SRR3124079). Reads were mapped using BWA with default parameters [50]. Samtools were used to exclude reads that were not uniquely mapped with the -q 20 parameter [51]. SNPs between B73, Mo17 and CAU5 inbred lines were called using Bcftools with default parameters [51]. Finally, we identified 508,700 SNPs covering 11,959 genes in the BM-C/C-BM and MB-C/C-MB crosses that could be used to distinguish parental alleles.

### 4.4. Measuring Allelic Expression and Identification of Imprinted Genes

To avoid bias, SNP sites were converted to CAU5 nucleotides to obtain the SNP-substituted CAU5 genome. All clean reads from three biological replicates of each sample were mapped to the B73 (Version 4) and SNP-substituted CAU5 genomes using HISAT2 with default parameters [48]. Samtools were used to exclude reads that were not uniquely mapped with the -q 20 parameter [51]. Three replicates from each sample were merged for the further identification of imprinted genes. According to the SNP information, the reads aligned at the SNP site were split into maternal or paternal alleles using Samtools mpileup. The maternal and paternal read counts of each gene were summed. If the summed read counts of annotated genes at all SNP sites were ≥20, the imprinting status of the gene could be analyzed. The ratio of maternal to paternal alleles of the analyzed genes was determined using the χ^2^ test to detect the deviation of the maternal: paternal ratio from the theoretically suggested 1:1 ratio in the embryo and 2:1 ratio in the endosperm. Finally, MEGs/PEGs were identified in the embryo if significant allelic bias (*p* value < 0.05; χ^2^ test) was detected and if >66% of the transcripts were derived from the maternal or paternal allele. In the endosperm, imprinted genes were identified as having significant allelic bias (*p* value < 0.05; χ^2^ test) if >80% of reads were from the maternal allele for MEGs or >50% of the reads were from the paternal allele for PEGs.

### 4.5. GO Term Enrichment and Functional Category Analysis

GO analysis of the imprinted genes was performed using Agri GO v2.0 [52]. Only GO terms among cell components, molecular functions and biological processes with significant (*p* value < 0.05) enrichment compared to all genes are displayed.

### 4.6. Validation of Imprinted Genes

We randomly tested the status of four SNPs in four imprinted genes detected in our study using a PCR sequencing method (*Zm00001d024959, Zm00001d020769, Zm00001d030305* and *Zm00001d032148*). Each gene fragment was amplified by different primers in eight 11-DAP embryo or endosperm cDNA samples: BB (self-cross of B73), MM (self-cross of Mo17), CC (self-cross of CAU5), BC and CB, MC and CM, BM-C and C-BM and MB-C and C-MB (Figure S10). The primer information is listed in Table S6.

### 4.7. Genetic Transformation of Maize

We prepared overexpression constructs for the genetic transformation of the non-conserved gene *Zm00001d020769* (*Zm769*). The full-length CDS (without the stop codon) of *Zm769* was amplified from *Zm769* cDNA and cloned into the binary vector pBCXUN-MYC to generate the pOE *Zm769*-MYC construct driven by the ubiquitin promoter. Transformations using the overexpression construct were introduced into the maize receptor line KN5585 via Agrobacterium-mediated transformation [53]. Independent positive transgenic lines were obtained and self-pollinated to generate homozygous progenies for kernel phenotype analysis.

### 4.8. Primers

All primers used in this study are listed in Table S6.

## 5. Conclusions

Imprinting variability exists in maize kernels and can contribute to observed parent-of-origin effects on kernel development. In this study, among the 192 and 181 imprinted genes identified in the BM-C/C-BM and MB-C/C-MB crosses, respectively, 51 genes were non-conserved, and specific GO terms were not enriched in these genes. Moreover, these genes were less conserved across other species. In addition, 11 high-confidence examples of allele-specific imprinting were discovered. Gene phenotypic analysis of one non-conserved PEG verified that the mutation of this gene made the embryo and kernel smaller than those of the wild type, and the overexpression of this gene enlarged the embryo and kernel area. Therefore, we concluded that non-conserved genes may also play an important role in kernel development, warranting more detailed functional analysis and further investigation of the mechanism of the non-conserved imprinted genes.

## Data Availability

Sequence data from this study can be found in the Sequence Read Archive at NCBI (SRA; http://www.ncbi.nlm.nih.gov/sra) under accession numbers PRJNA874824 and PRJNA765150.

## Supplementary Materials

The following supporting information can be downloaded at: https://www.mdpi.com/article/10.3390/ijms232315424/s1.

## Abbreviations

BB represents the self-cross of B73; MM represents the self-cross of Mo17; CC represents the self-cross of CAU5; BM and MB represent the crosses of B73 × Mo17 and Mo17 × B73, respectively; BC and CB represent the crosses of B73 × CAU5 and CAU5 × B73, respectively; MC and CM represent the crosses of Mo17 × CAU5 and CAU5 × Mo17, respectively; BM-C represents the crosses of (B73 × Mo17) × CAU5; C-BM represents the crosses of CAU5 × (B73 × Mo17); MB-C represents the crosses of (Mo17 × B73) × CAU5; C-MB represents the reciprocal crosses of CAU5 × (Mo17 × B73); DAP: Days after pollination; MEGs: Maternally expressed genes; PEGs: Paternally expressed genes; WT: Wild type.
