# Supplementary material for: Conservation Study of Imprinted Genes in Maize Triparental Heterozygotic Kernels
Source: Int J Mol Sci. 2022 Dec 6;23(23):15424. doi: 10.3390/ijms232315424 (PMC9735609; doi:10.3390/ijms232315424)
Supplement: Supplementary file 1 [file ijms-23-15424-s001.zip › Supplemental Figures.pdf]

A
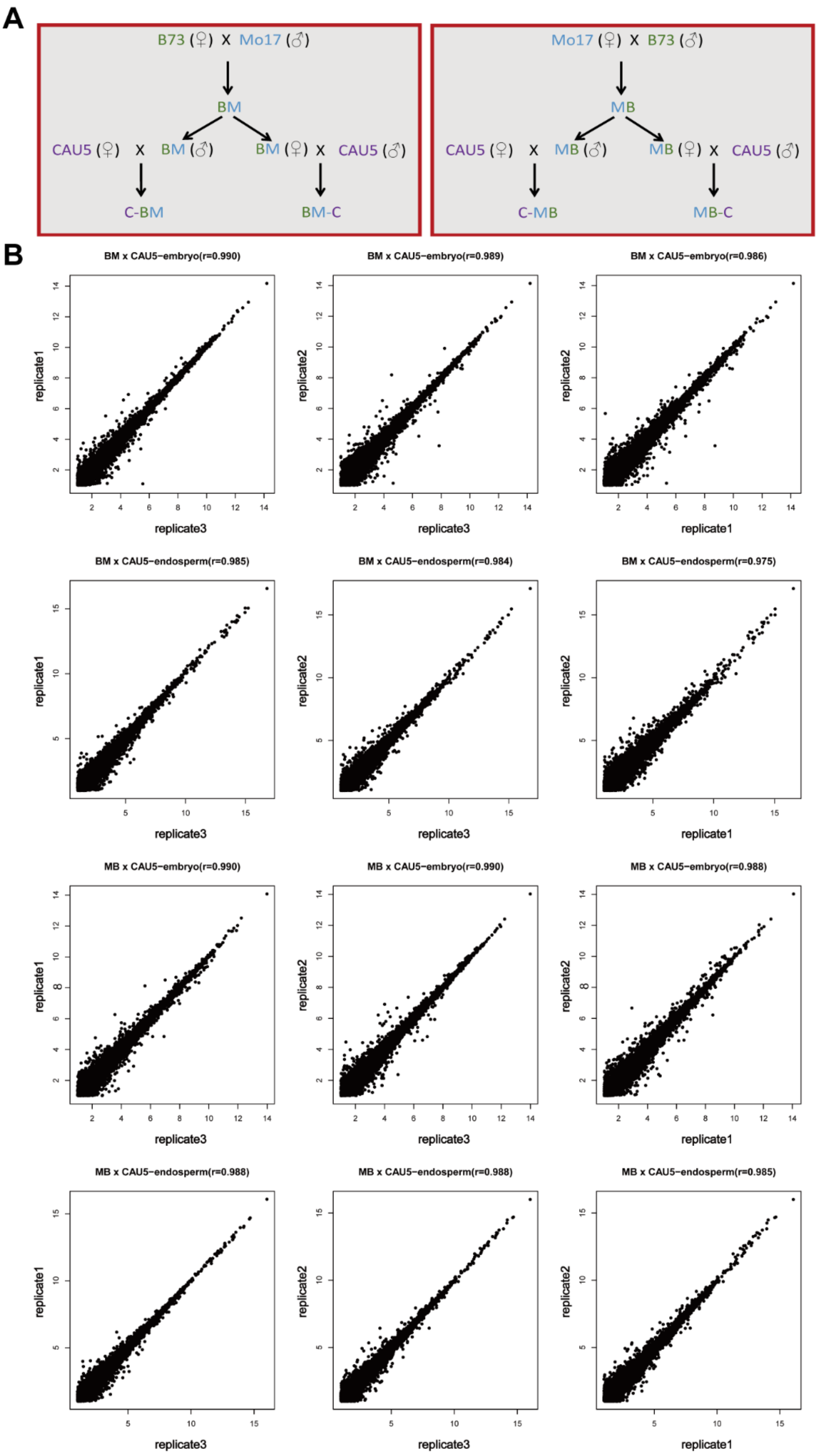

Supplemental Figure S1 The cross progress for BM-C/C-BM and MB-C/C-MB (A) and the correlation among replicates of embryo and endosperm samples from BM-C/ C-BM and MB-C/C-MB (B).

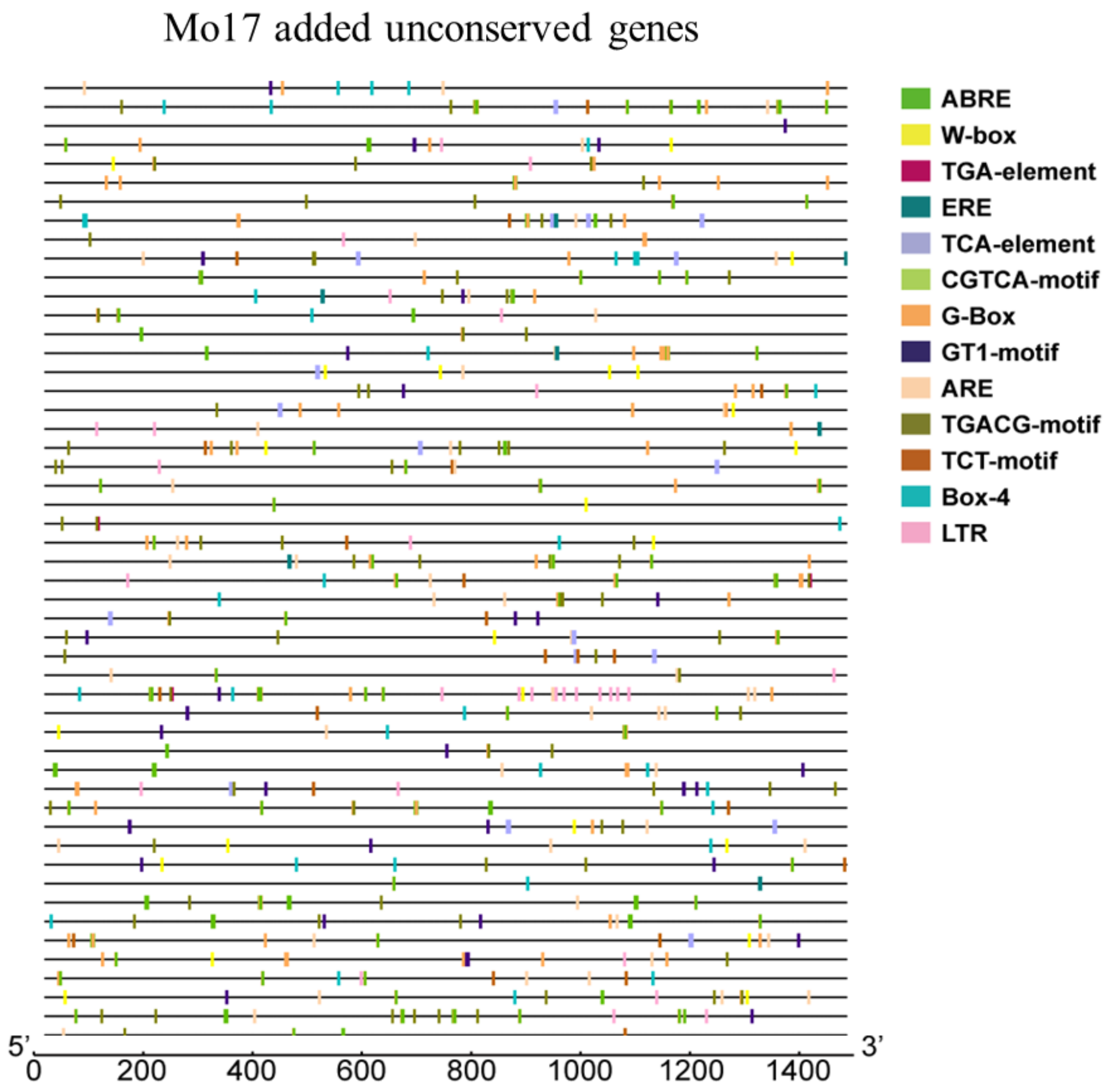

Supplemental Figure S2 The cis-element distribution in the promoter sequence of the 51 non-conserved imprinted genes detected in the Mo17-added group

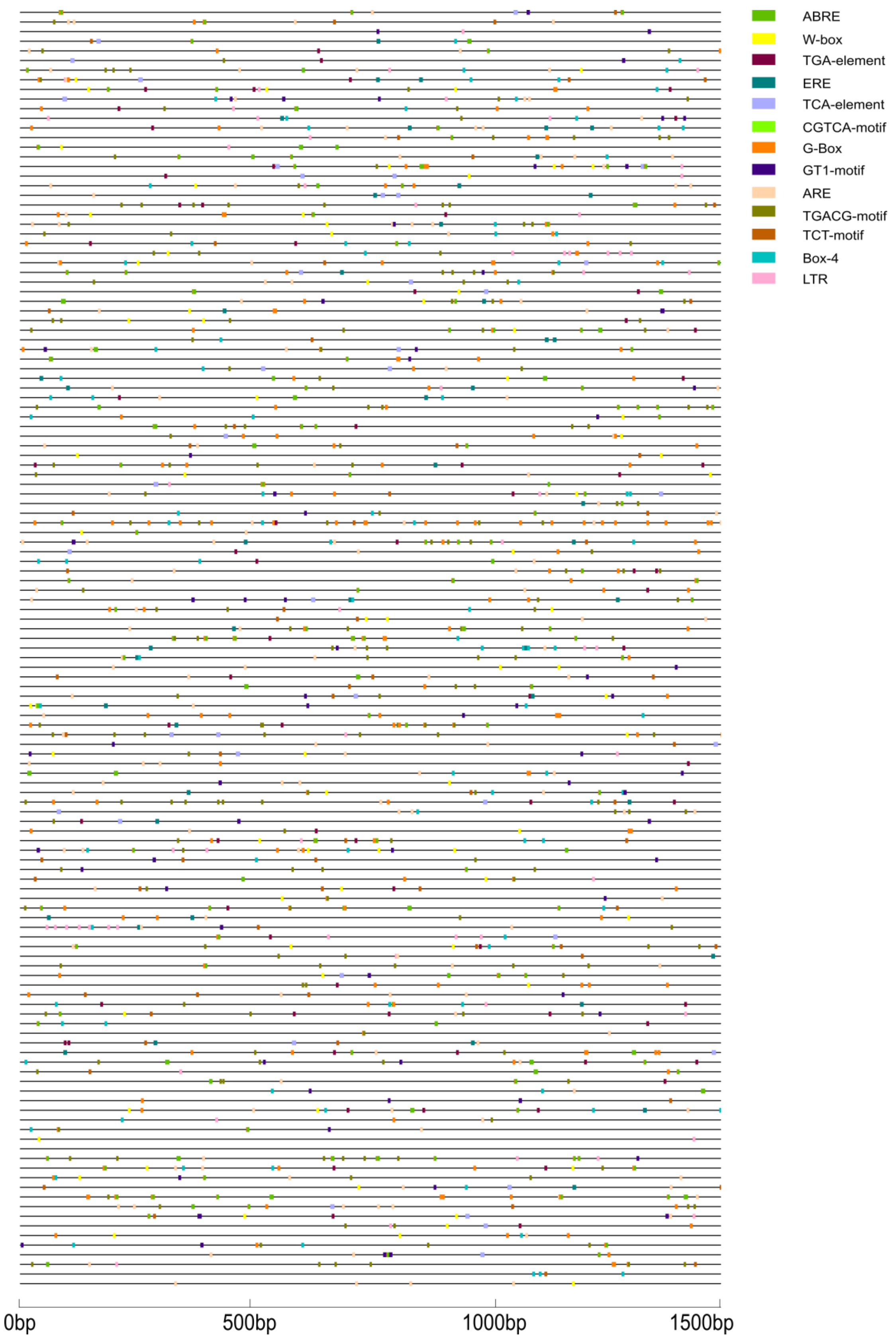

Supplemental Figure S3 The cis-element distribution in the promoter sequence of the conserved imprinted genes detected in the B73-added group

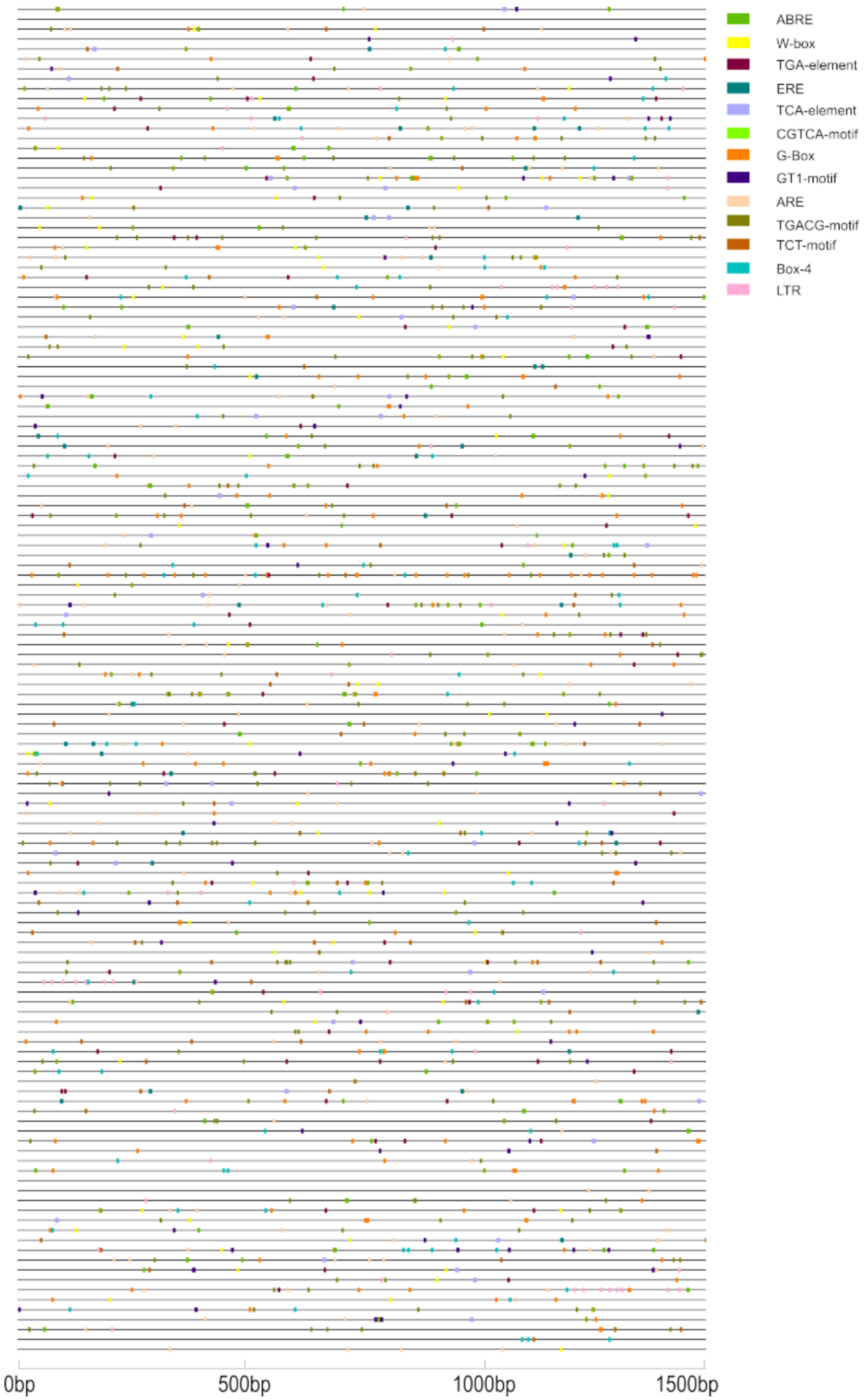

Supplemental Figure S4. The cis-element distribution in the promoter sequence of the conserved imprinted genes detected in the Mo17-added group

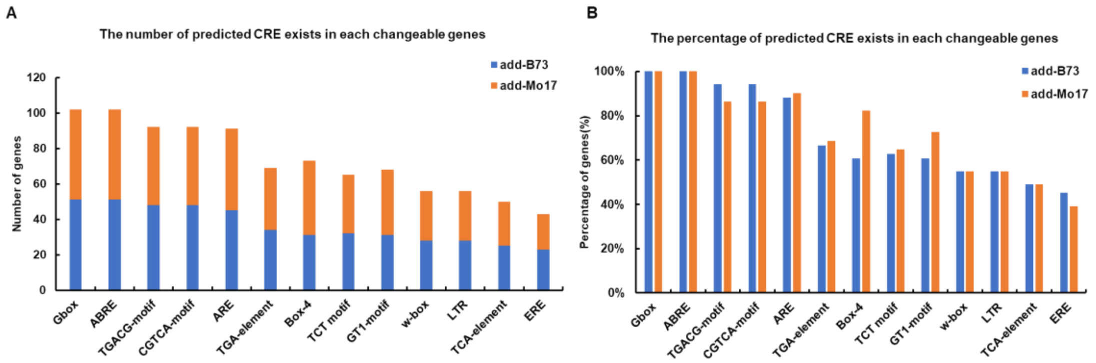

Supplemental Figure S5. Distribution of predicted CREs in each non-conserved gene

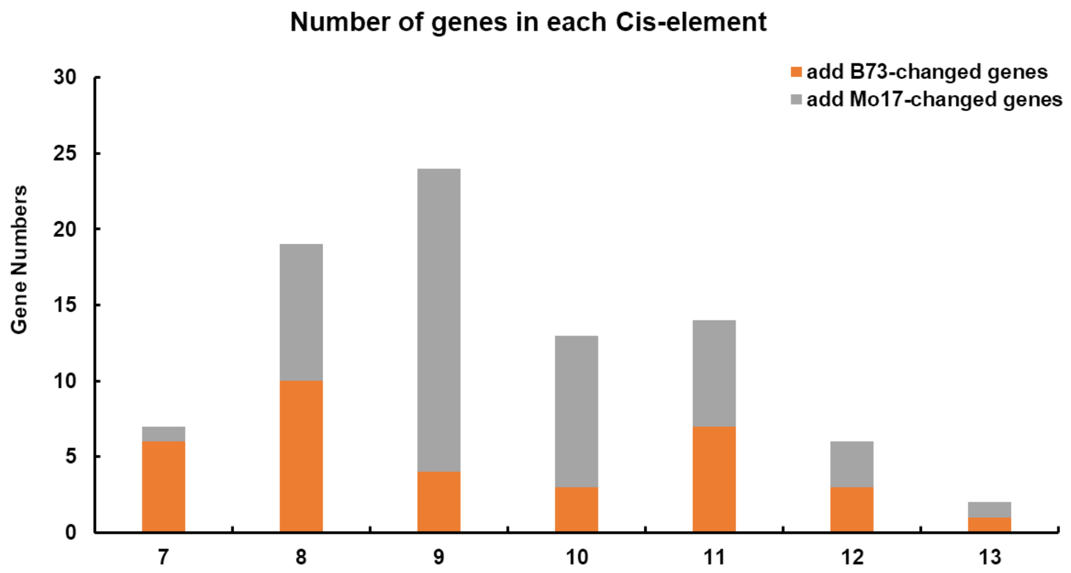

Supplemental Figure S6. The ratio of each Cis-element existed in two groups of non-conserved genes

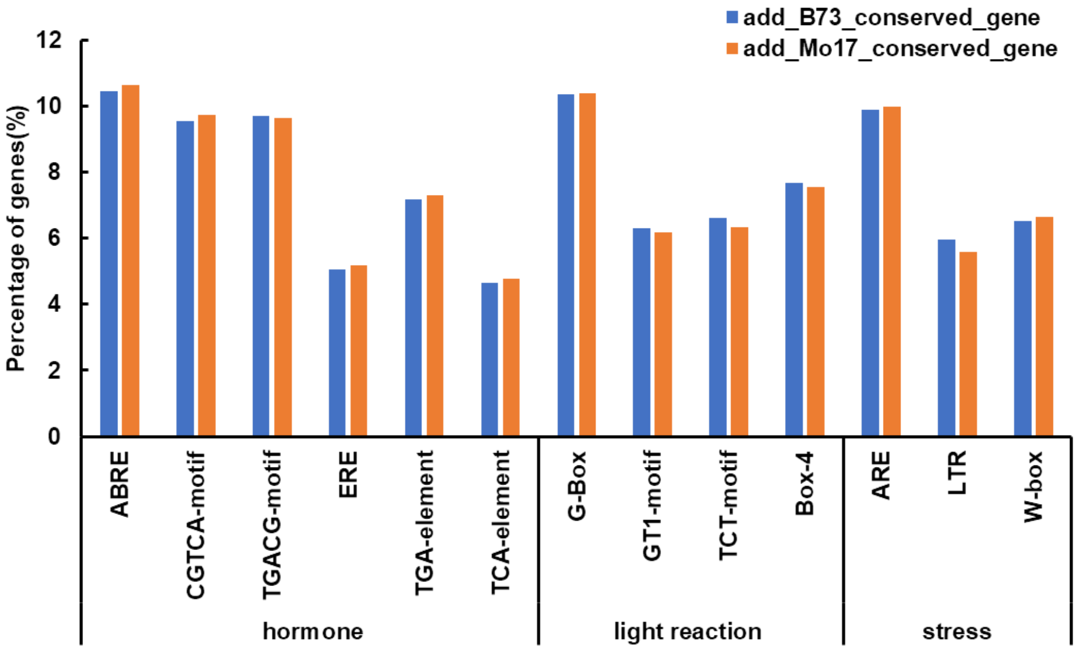

Supplemental Figure S7. The ratio of each Cis-element existed in two groups of conserved genes

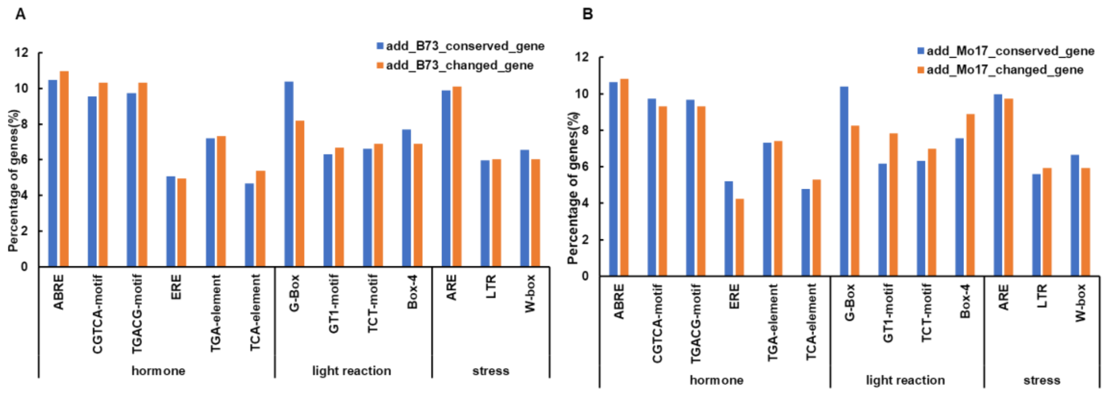

Supplemental Figure S8. Ratio comparison of each cis-element in the conserved and non-conserved genes in the B73-added and Mo17-added groups

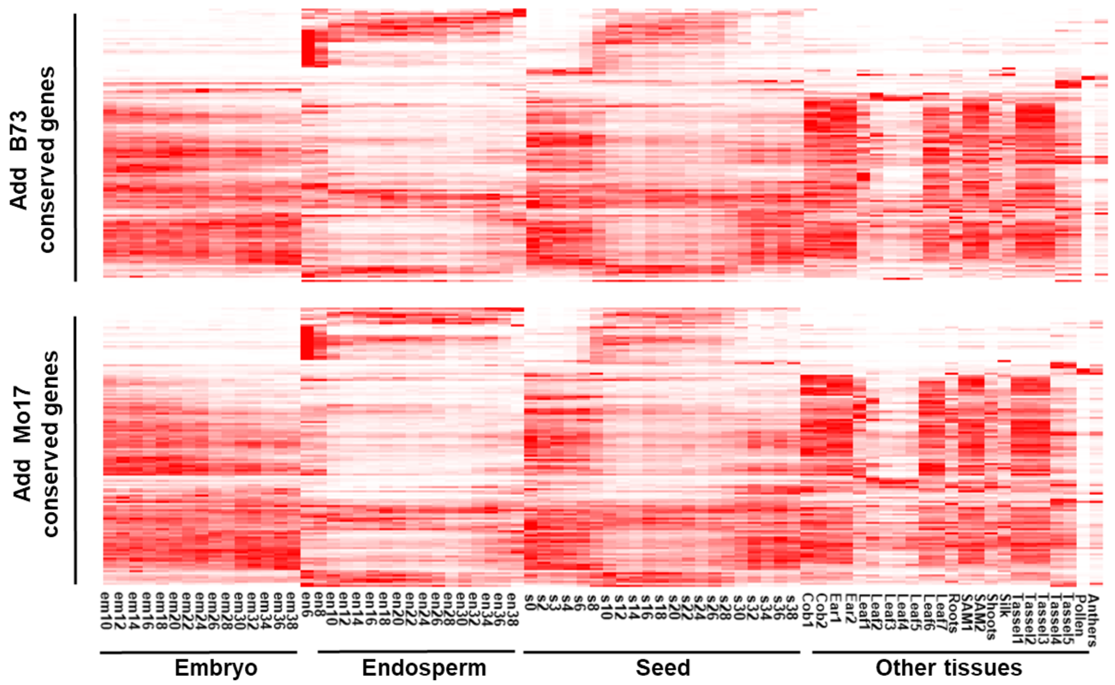

Supplemental Figure S9. The expression of conserved imprinted genes detected in the B73-added and Mo17-added groups

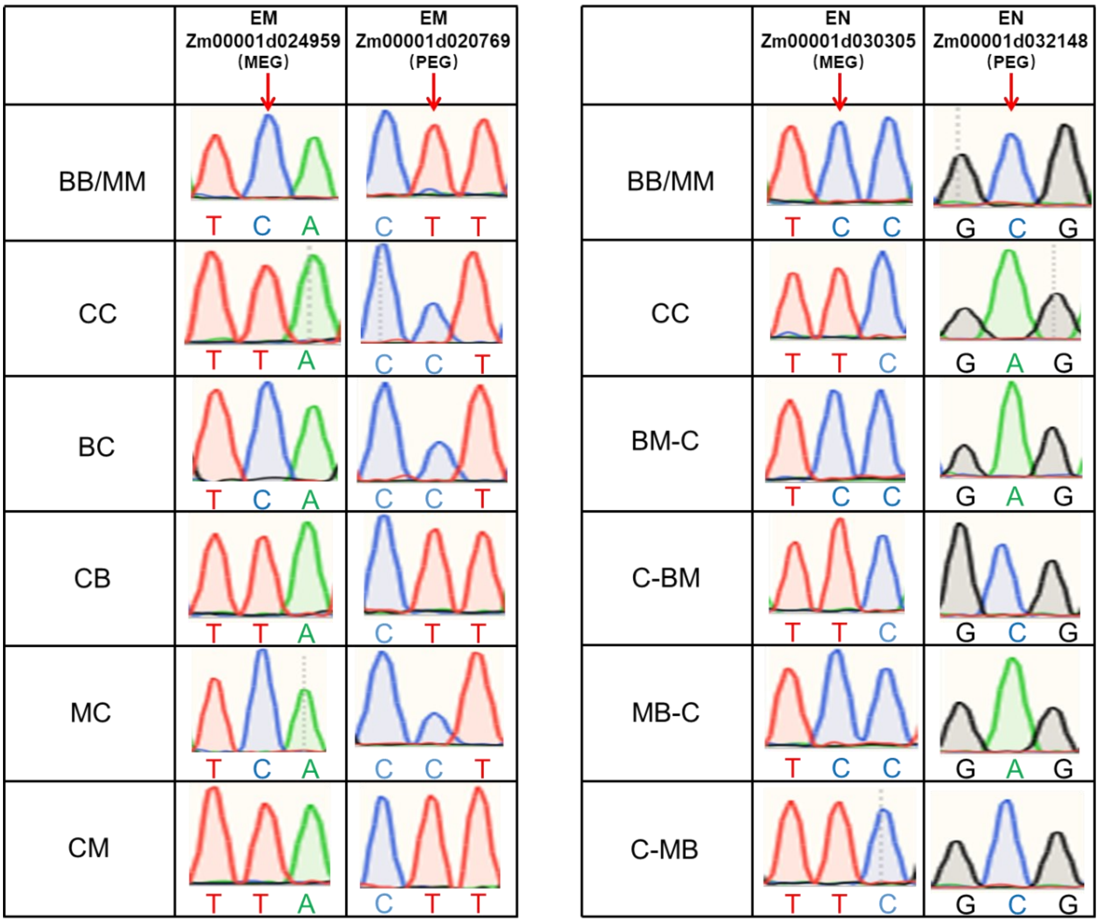

Supplemental Figure S10. Verification of the imprinted genes by PCR sequencing
